# Migraine and possible etiologic heterogeneity for hormone-receptor-negative breast cancer

**DOI:** 10.1038/srep14943

**Published:** 2015-10-12

**Authors:** Min Shi, Lisa A. DeRoo, Dale P. Sandler, Clarice R. Weinberg

**Affiliations:** 1 Biostatistics & Computational Biology Branch, NIEHS, NIH, DHHS, Research Triangle Park NC; 2 Epidemiology Branch, NIEHS, NIH, DHHS, Research Triangle Park; 3 Department of Global Public Health and Primary Care, University of Bergen, Bergen, Norway

## Abstract

Migraine headache is often timed with the menstrual cycle. Some studies have reported reduced risk of breast cancer in migraineurs but most of those did not distinguish menstrually-related from non-menstrually-related migraine. To examine the possible associations between breast cancer and migraine overall and between cancer subcategories and the two migraine subtypes, we used a cohort study of 50,884 women whose sister had breast cancer and a sister-matched case-control study including 1,418 young-onset (<50 years) breast cancer cases. We analyzed the two studies individually and also in tandem via a hybrid Cox model, examining subcategories of breast cancer in relation to menstrually-related and non-menstrually-related migraine. History of migraine was not associated with breast cancer overall. Migraine showed an inverse association with ductal carcinoma *in situ* (HR = 0.77; 95% CI (0.62,0.96)). Also, women with non-menstrually-related migraine had increased risk (HR = 1.30, 95% CI (0.93,1.81)) while women with menstrually-related migraine had decreased risk (HR = 0.63, 95% CI (0.42,0.96)) of hormone-receptor-negative (ER−/PR−) cancer, with a significant contrast in estimated effects (*P* = 0.005). While replication of these subset-based findings will be needed, effect specificity could suggest that while migraine has little overall association with breast cancer, menstrual migraine may be associated with reduced risk of ER−/PR− breast cancer.

Migraine headache is a common and chronic disorder that occurs more often in women than men^1^. Slightly more common in girls than in boys, after puberty the prevalence of migraine increases in females, rising to a peak in the early 40 s^1^. The rate of new diagnoses declines after menopause^2^, after which prevalence also declines^3^.

Migraine can be sensitive to hormones. Approximately 50% of women with migraine report that attacks tend to be synchronized with their menstrual cycle^4^. The typical pattern is that headache begins within a day or two before menses onset, during the fall of estrogen at the end of the cycle. Randomized placebo-controlled clinical trials have suggested some prophylactic therapeutic benefit from estrogen therapy for menstrual migraine, consistent with hormone sensitivity^5^,^6^.

Breast cancer is also related to hormones: higher rates are seen in women with early age at menarche, late age at first birth, and late age at menopause^7^,^8^. The use of peri- and postmenopausal hormone replacement therapy can increase the risk of breast cancer^9^,^10^. A positive association between endogenous levels of blood estrogen and androgen and postmenopausal breast cancer has been reported in several studies^11^,^12^,^13^, and a recent nested case-control study within the Nurses’ Health Study showed that a single blood-based sex hormone measurement predicted breast cancer risk even 16–20 years later^14^. Tumors are categorized according to whether they express hormone receptors and consequently are hormone sensitive or not, a difference with implications both for treatment and prognosis^15^.

An association between migraine and breast cancer risk has been hypothesized. Several studies reported an inverse association, with 10–30% reduction in risk in women with a migraine history^16^,^17^,^18^. This inverse association was not replicated by a recent analysis based on the Women’s Health Study^19^. None of the studies distinguished menstrually-related from other migraine. We used data from the Sister Study cohort of 50,884 women whose sister had breast cancer, and the Two Sister Study, a sister-matched case-control study of 1,418 breast cancer cases diagnosed before age 50. We analyzed the two studies separately and also in combination. We examined subcategories of breast cancer (invasive breast cancer versus ductal carcinoma *in situ* (DCIS), estrogen-receptor/progesterone-receptor (ER/PR) positive versus negative, and hormone receptor negative and HER2 negative (triple negative) versus others) in relation to menstrually-related and other migraine. Our objective was to determine whether migraine *per se* was associated with the risk of breast cancer and whether that association differed by tumor subtype or was modified by whether or not the migraine was menstrually related. We took advantage of two large studies that had used very different designs: one (Sister Study) was prospective, population-based and required a first-degree family history, while the other (Two Sister Study) was retrospective, family-based and included only about 20% with a first-degree family history. Because the two could be analyzed in a way that ensured statistical independence of findings, we were able to assess reproducibility of findings.

## Methods

### The two studies

The Sister Study recruited 50,884 women in the United States and Puerto Rico (http://sisterstudy.niehs.nih.gov/English/about.htm), who were age 35 to 74 at enrollment in 2004–2009 and had a sister with breast cancer, but had never been diagnosed with breast cancer themselves^20^. Participants provided information on demographic and lifestyle characteristics, family history, medical conditions, reproductive history, and occupational and environmental exposures. Incident breast cancer cases were identified over a mean follow-up of 5.34 years, based on annual health updates and biennial/triennial questionnaires and confirmed through additional participant contact and retrieval of medical records.

The Two Sister Study (http://www.sisterstudy.niehs.nih.gov/English/2sis.htm) is a family-based retrospective case-control study ancillary to the Sister Study. With the help of already-identified Sister Study participants, we enrolled case sisters who had been diagnosed recently (within 4 years) and under age 50. Enrollment took place from September 2008 to December 2010. Both the Sister Study and the Two Sister Study secured informed consent and were carried out with human subjects approval and oversight from the NIEHS Institutional Review Board and the Copernicus Group Institutional Review Board. Both studies were carried out in accordance with the approved guidelines.

Participants all completed the same risk factor questionnaires. In addition, cases provided details on their breast cancer diagnosis, tumor characteristics, and treatment, and they authorized access to medical records. Tumor subtypes (ER, PR, and human epidermal growth factor receptor 2 (HER2)) were ascertained from medical records (80%–89%) when available, or from self-report (11%–20%). Agreement between these two sources was excellent^21^. We also carried out exploratory analyses based on subcategorizing invasive cancer by histologic type as lobular or ductal.

The baseline questionnaire included the question: “Has a doctor or other health professional ever told you that you had migraine headaches?” Those who responded “Yes” were then asked the age at which that diagnosis had been made. Responses to the additional question “Have you ever noticed a pattern where your migraine headaches got worse at certain times of your menstrual cycles?” were used to classify migraine as menstrual versus nonmenstrual. Women with both types were categorized as having menstrual migraines. We also collected data on use of migraine medications, for sensitivity analyses. Data were not collected on family history of migraine.

### Statistical Analysis

Data from the prospective Sister Study cohort was modeled using Cox proportional hazards regression, with age as the primary time scale. Follow-up began at enrollment (or to avoid overlap, for Two Sister controls follow-up began at the age at which their proband sister was diagnosed, if that was later) and ended at breast cancer diagnosis or (for women remaining without breast cancer) age at most recent follow-up. For assessing associations with specific subcategories of breast cancer, diagnoses of other types or of unknown subcategory were treated as censoring events that truncated time at risk.

Cox proportional hazards regression is equivalent to conditional logistic regression with fine stratification on ages at events, and data from the Two Sister Study were also analyzed with conditional logistic regression, but with additional stratification on sibship. For many families the matched control sister was older than their case sister, because eligible case sisters had to have been under age 50 at diagnosis by design. We used “index age” to ensure comparable opportunity for time-dependent exposures in within-sibship comparisons, as previously described^21^. Briefly, index age was defined for each set of sisters as the smallest of the reported age of the case sister at diagnosis and the age(s) of her control sister(s) at completion of enrollment. For example, if the case sister was diagnosed at 48 and her control sister enrolled at age 50, the index would be 48 for the sister pair. The exposure experienced by the control sister between 48 and 50 was not considered to be relevant. For most sibships (N = 893) the index age was the same as the age at diagnosis and for others it was somewhat younger. For Two Sister Study analyses women were categorized as having migraine only if their stated age at migraine diagnosis was earlier than their sibship’s index age. (Migraine tends to be diagnosed early in life and few (N = 54) had been diagnosed with migraine after the index age, but to ensure comparable opportunity to have had a migraine diagnosis, such women were not considered to have migraine in our analyses.) The use of index age in this way accomplishes age adjustment in the analysis of the Two Sister Study.

We also carried out completely separate analyses and used meta-analyses to combine the results of the separately-analyzed two studies (with inverse variance weighting) to confirm that the point estimates based on combined-analysis and meta-analysis were similar.

Despite the fact that some of those followed in the Sister Study also served as controls for the Two Sister Study, the two studies provide statistically independent inference. This independence arises from the fact that the overall likelihood is the product of the two likelihoods. The Cox model (as with all life table methods) treats outcomes at different event times as statistically independent (under a Markov assumption), and controls are used over and over again in the same analysis, until they become cases. One consequence is that the overall likelihood is the product of many separate likelihoods, one for each event time. The control sister for the Two Sister Study was eligible to be a control because she was free of cancer at the age when her sister was diagnosed. Her own subsequent experience with breast cancer risk in the Sister Study follow-up was analyzed prospectively. To ensure that the two studies contributed independently to the analysis, we started the clock for the Sister Study likelihood either at the participant’s age at completion of enrollment or, if she was also serving as a control for Two Sister, at the age when her proband sister was diagnosed, whichever was later. Thus the two designs are separable and provide non-overlapping, statistically independent inference.

Migraine history was coded as either a binary variable or a three-level variable (no migraine, nonmenstrually-related, and menstrually-related migraine). A small number of women who did not specify menstrually-related or not (1.2% of total migraine reporters) were treated as nonmenstrually-related, presuming that a relationship with menses would have been noticed. We separately evaluated association with invasive breast cancer and ductal carcinoma *in situ* (DCIS). The invasive cancer subgroup analyses were further divided into tumor-based estrogen receptor/progesterone receptor (ER/PR) status subtypes. Cases missing ER/PR status were treated as censored at diagnosis for the Sister Study and were excluded for Two Sister. We adjusted for the following potential confounders identified by consideration of directed acyclic graphs (DAG)^22^: race, age at menarche, nulliparity, body mass index (BMI) age at first term birth for parous women and menopausal status. We assessed heterogeneity between menstrually-related and nonmenstrually-related migraine by using likelihood ratio tests of the null hypothesis that the migraine coefficients are the same. We also explored effect modification by age at risk, menopause status and BMI as recalled for when they were in their 30 s via likelihood ratio testing. We also conducted case-only logistic analysis^23^,^24^, which included all cases, to examine etiologic heterogeneity across cancer subcategories, adjusting for race, age at diagnosis, age at menarche, study, and age at first birth. As a spline model did not improve the fit, age at diagnosis was included linearly in case-only analyses.

All analyses were done using the SAS software, PHREG and LOGISTIC procedures, version 9.3 (SAS Institute, Inc., Cary, NC). All statistical tests were two-sided.

## Results

Similar percentages of women in the Sister (20.7%) and Two Sister (18.5%) studies reported migraine history and among them similar percentages, 44.5% (Sister) and 45.5% (Two Sister), reported their migraine tended to be timed to the menstrual cycle, referred to as menstrually-related migraine. Table 1 shows characteristics of participants in the two studies stratified by migraine status. The mean ages of women without migraine and those with nonmenstrually-related migraine in the Sister Study were similar (56 years) while those reporting menstrually-related migraine were slightly younger (mean age = 54 years). The mean index age of the Two Sister Study participants was 44 and did not depend on migraine status. The mean age of control sisters was 47.3 (standard deviation [SD] = 6.3 years) at enrollment and the mean age of case sisters was 47.3 (SD = 4.1 years) at enrollment, and 44.7 (SD = 4 years) at diagnosis. Overall, characteristics were similar for participants across the two migraine categories in both studies, except that women with menstrual migraine were more often pre-menopausal at interview. During a mean follow up of 5.3 years (270,866 person-years), 2,118 newly incident breast cancer cases were reported in the Sister Study, which included 1,534 invasive and 488 DCIS cases. The Two Sister Study retrospectively included 1,200 invasive and 203 DCIS cases.

As shown in Table 2, neither category of migraine was significantly associated with breast cancer overall [combined-analysis adjusted HR = 1.03 (0.90, 1.18) for nonmenstrually-related migraine, HR = 0.91 (0.77, 1.07) for menstrually-related migraine] or with invasive breast cancer [combined-analysis adjusted HR = 1.07 (0.93, 1.22) for nonmenstrually-related migraine, HR = 0.94 (0.80, 1.10) for menstrually-related migraine]. An inverse association between undifferentiated migraine and DCIS was observed in the Sister Study (adjusted HR = 0.73 (0.57, 0.94) for any migraine) and in the combined analysis, but was not seen in the Two Sister Study (adjusted OR = 1.04 (0.61, 1.79). The association with DCIS in the Sister Study was similar for nonmenstrually-related (adjusted HR = 0.73 (0.53, 1.01) versus menstrually-related (adjusted HR = 0.73 (0.51, 1.05)) migraine.

Migraine was not associated with ER+/PR+ invasive cancer in either study (combined-analysis adjusted HR = 1.03 (0.86, 1.23) for nonmenstrually-related migraine, HR = 1.04 (0.86, 1.27) for menstrually-related migraine) (Table 2). For invasive ER−/PR− cancer, there was again no overall association between migraine and risk (combined-analysis adjusted HR = 0.96 (0.73, 1.26)). The meta-analysis results were similar to the combine-analysis results (adjusted HR = 0.95 (0.72, 1.25)). However, women with nonmenstrually-related migraine had some evidence for increased risk (combined-analysis adjusted HR = 1.3 (0.93, 1.81)) while women with menstrually-related migraine had decreased risk (combined-analysis adjusted HR = 0.63 (0.42, 0.96)). The difference was statistically significant (*P* = 0.005) and the same contrasting effect directions were seen in both of the two studies. Again the meta-analysis results were similar to the combined-analysis results (adjusted HR = 1.3 (0.93, 1.82) and 0.61 (0.40, 0.94) for menstrually-related and nonmenstrually-related migraine, respectively). Similar associations were observed for the subcategory of invasive ER−/PR−/HER2− cancer (Table 2). We did not observe effect measure modification by age at risk (≥45 years or <45), menopause status or by body mass index based on recalled average nonpregnant weight during their 30 s (data not shown). In a sensitivity analysis, we also fit a model adjusting for the known risk factors for breast cancer: race, age at menarche, age at first term birth, parity, menopause status, BMI in the 30 s, birth control use, hormone replacement therapy use, alcohol and smoking. Results were similar to those based on only adjusting for potential confounders identified by consideration of directed acyclic graphs (DAGs).

In a case-only analysis of invasive breast cancer modeling the odds of ER negative cancer, conditional on cancer having occurred, nonmenstrually-related migraine was positively associated with estrogen-receptor negative (ER−) cancer whereas menstrually-related migraine was negatively associated with estrogen-receptor negative (ER−) cancer (combined-analysis test of heterogeneity *P* = 0.06) (Table 3). In a model for the odds of ER−/PR− cancer, conditional on cancer having occurred, cases with nonmenstrually-related migraine were more likely to have ER−/PR− cancer (combined-analysis OR = 1.26 (0.99, 1.60)) while cases with menstrually-related migraine were less likely to have ER−/PR− cancer (combined-analysis OR = 0.78 (0.59, 1.03), combined-analysis test of heterogeneity *P* = 0.044). The risk for other subcategories or DCIS versus non-DCIS did not differ appreciably by migraine type.

In a sensitivity analysis, we only categorized women as having migraine if they reported both a positive migraine history and a history of use of migraine medication. Similar, but slightly stronger, associations for DCIS and ER−/PR− cancers were observed with this stricter migraine definition (data not shown). We reanalyzed our data focusing on histological subtypes and did not find any association in either ductal or lobular invasive breast cancer. (Data not shown.).

## Discussion

We analyzed a sister-matched case-control study and a cohort study and neither showed an overall association between self-reported migraine headache and breast cancer. There was evidence for an inverse association between migraine and DCIS in the cohort study, and in the combined analysis, but limited evidence for that association in the Two Sister Study. However, the Two Sister Study included few cases with DCIS, and there was considerable overlap in the confidence intervals, indicating no important inconsistency between the two studies.

We considered whether medical screening bias could be a factor, since the Sister Study cohort participants all have a first-degree family history of breast cancer, while the majority of cases in the retrospective Two Sister Study did not have a first-degree family history. Women with a sister with breast cancer who themselves have migraine may be more likely, because of their own chronic condition, to have regular medical care and hence also to be screened for breast cancer. However, instead of an inverse association, one would expect to see a positive association between migraine and DCIS if there were screening bias in the Sister Study, whereas the association we saw was negative.

Migraine, like breast cancer, is an estrogen-sensitive disorder that primarily affects women^25^. The sensitivity of ER+ breast tumors to estrogen has enabled the development of effective therapeutic regimens, but hormone-receptor negative tumors remain a clinical challenge.

When we subcategorized migraine as menstrually-related versus nonmenstrually-related, and considered hormone-receptor-negative breast cancer, patterns emerged that were similar in the two studies. We saw increased estimated risk for hormone-insensitive ER−/PR− breast cancer in women reporting nonmenstrually-related migraine and decreased risk in women reporting menstrually-related migraine. Thus data from both studies are consistent with the possibility that women with hormone-insensitive migraine are at relatively increased risk for the more aggressive hormone-receptor-negative breast cancer.

The existing literature on migraine and breast cancer is mixed. Three case-control^17^,^18^,^26^ and three prospective cohort studies^16^,^19^,^27^ previously examined the association between migraine and breast cancer. Four of them^16^,^17^,^18^,^26^ reported a negative association while two found none^19^,^27^. Mathes *et al.*^18^, Li *et al.* (2010)^16^ and Lowry *et al.*^26^ studied postmenopausal women only, while Li *et al.* (2009)^17^ and Winter *et al.* (2013)^19^ studied both premenopausal and postmenopausal women and found no difference between them in the association between migraine history and risk. Lowry *et al.*^26^ used a subset of the subjects in Mathes *et al.*^18^ and obtained more detailed information on migraine history for a more refined analysis. They observed that women with early onset migraine or long-duration migraine history had a reduced risk of ER+ breast cancer. In addition to invasive breast cancer, Li *et al.* (2010)^16^ also studied *in situ* breast cancer and found an inverse association with migraine, which was not statistically significant but is consistent with our finding. However, Winter *et al.*^19^,^27^ found no association with DCIS. Winter *et al.* (2015)^27^ performed a meta-analysis based on these six previous studies and provided a nice summary of the studies in their Supplementary Table 2.

Previous studies that did not categorize migraine by hormone sensitivity showed diverse results regarding the association between migraine and ER−/PR− breast cancer risk. An inverse association was reported by Mathes *et al.* (OR = 0.87 (0.56–1.36))^18^ and Li *et al.* (2009) (OR = 0.83 (0.70–0.99))^17^ while a positive association was reported by Li *et al.* (2010) (OR = 1.16 (0.86, 1.57))^16^ and Winter *et al.* (OR = 1.28 (0.96, 1.71))^19^. In the Nurses’ Health Study II, the OR for ER−/PR− breast cancer was close to one (OR = 0.93 (0.73, 1.17)^27^. Previous studies also investigated the association by histologic subtype and reported similar effects for ductal and lobular carcinoma.

In our analysis, an association of migraine with breast cancer was not seen in general. However, with further exploration a relationship with ER−/PR− cancer was seen when we categorized migraine by hormone sensitivity. This observation, based on subcategories, would require replication. Among the six previous publications, only Lowry *et al.*^26^, in carrying out a subset analysis of the subjects in Mathes *et al.*^18^, obtained more detailed information on migraine history distinguishing menstrually-related from nonmenstrually-related migraine. However, they did not specifically study ER−/PR− cancer but reported results for ER− cancer. Their case sample size was small for ER- cancer but their reported contrast in ORs was consistent with our findings (OR = 1.8 for nonmenstrually-related migraine and OR = 0.5 for menstrually-related migraine).

Some limitations of our study are worth mentioning. Information on migraine history was self-reported and some misclassification may exist. Nevertheless, a re-analysis of the data by categorizing women as having migraine only if they also reported use of migraine medication produced similar results to those based on the original self-reported migraine status.

We also have used self-reported information on whether their migraine attacks tended to be timed with the menstrual cycle, as “menstrually-related migraine,” rather than the stricter category of “menstrual migraine” defined by the International Headache Society^28^. It would have been interesting to see whether a stricter classification might have strengthened the apparent risk contrast that we found. We also were unable to distinguish between women with only menstrual migraine and women with both types, nor did we have access to medical records related to migraine. However, medical records would likely also need to rely on the woman’s self-report. The frequency of reporting menstrually-related migraine fell off after menopause in our study, suggesting that some postmenopausal participants may have mistakenly assumed we were asking about their current pattern. Consistent with that possible explanation is the observation that when we exclude Sister Study women who had completed the questionnaire more than twelve years after their menopause, the results for ER−/PR− invasive cancer become somewhat stronger than those shown in Table 2: HR = 0.58; CI = (0.3, 1.15) for menstrually-related migraine and HR = 1.55; CI = (1.01, 2.37) for nonmenstrually-related migraine.

Finally, for the Sister Study we used information collected at baseline, without updating it during follow-up. However, considering that 93% of migraine cases are diagnosed before age 50 2 and our follow-up time is limited (averaging 5.34 years) few women would have developed migraine during follow up. A sensitivity analysis that limited follow-up to the first 3 years after enrollment produced very similar results.

Our study has several strengths. One is that the findings with respect to ER−/PR− breast cancer were reproduced in two studies with very different designs (Table 2): a large prospective cohort of women with a first-degree family history of breast cancer and a retrospective family-based case-control study of people, most of whom did not have a family history. The same information on confounders was available in both studies and the two studies provided statistically independent and similar findings relating migraine to ER−/PR− breast cancer.

In summary, using data from a prospective cohort study and a sister-controlled retrospective study of breast cancer we found evidence that migraine is negatively associated with DCIS. However, the DCIS association was based on the Sister Study cohort and was not observed in the Two Sister Study.

When attention was turned instead to invasive ER−/PR− breast cancer, we identified statistically significant heterogeneity in risk in women with nonmenstrually-related versus menstrually-related migraine, with the same pattern evident in both studies. This observation, while needing future confirmation, suggests that women with hormone-insensitive migraine who develop breast cancer may be more likely to produce a hormone-insensitive ER−/PR− tumor. The inverse observation that a history of menstrually-related migraine appears to be protective against ER−/PR− breast cancer has now been noted twice (our combined and that of Lowry *et al.*^26^).

These could be chance findings and one should generally be wary of inferences based on subcategories of the predictor and sub-categorizations of the outcome. The underlying biology that would explain the observed disparate relationships is not clear. To speculate, we think it is unlikely that hormone-sensitive tumors are caused by hormone-sensitive migraine and prevented by hormone-insensitive migraine. A more parsimonious explanation would be that some women, perhaps based on genetic predisposition, tend to express hormone receptors and they tend to do so both in connection with migraine and as part of the phenotype when they develop a breast tumor. Our findings will need to be replicated in future studies, but such a phenotype-modifying mechanism would be consistent with the absence of an overall effect of migraine on risk of breast cancer.

## Additional Information

**How to cite this article**: Shi, M. *et al.* Migraine and possible etiologic heterogeneity for hormone-receptor-negative breast cancer. *Sci. Rep.* **5**, 14943; doi: 10.1038/srep14943 (2015).

## Figures and Tables

**Table 1:** Selected characteristics by migraine status and study.

Characteristic	Sister Study*			Controls in Two Sister Study*		
	No Migraine (n = 40154)	Nonmenstrual Migraine (n = 5807)	Menstrually-related Migraine (n = 4651)	No Migraine (n = 1353)	Nonmenstrual Migraine (n = 168)	Menstrually-related Migraine (n = 140)
Mean age at baseline						
	55.9	55.8	53.9	47.9	46.9	46.9
**Mean index age**						
	—	—	—	43.5	43.6	43.5
**Race**						
Non-Hispanic white	33536 (84)	4843 (83)	3959 (85)	1206 (89)	150 (89)	126 (90)
Black	3759 (9)	499 (9)	318 (7)	66 (5)	3 (2)	6 (4)
Hispanic	1822 (5)	288 (5)	252 (5)	48 (4)	10 (6)	3 (2)
Other	1026 (3)	173 (3)	122 (3)	32 (2)	5 (3)	5 (4)
**Education**						
High school or less	6230 (16)	919 (16)	631 (14)	184 (14)	21 (13)	9 (6)
Some college but no degree	7687 (19)	1299 (22)	941 (20)	221 (16)	32 (19)	27 (19)
Associate or technical degree	5605 (14)	841 (14)	731 (16)	209 (15)	27 (16)	15 (11)
Bachelor degree	10943 (27)	1438 (25)	1242 (27)	425 (31)	42 (25)	56 (40)
Master or doctoral degree	9689 (24)	1310 (23)	1106 (24)	314 (23)	46 (27)	33 (24)
**Age at menarche**						
<12 y	8025 (20)	1334 (23)	1037 (22)	214 (16)	33 (20)	28 (20)
12-<14 y	22654 (56)	3149 (54)	2565 (55)	786 (58)	86 (51)	82 (59)
>=14 y	9475 (24)	1324 (23)	1049 (23)	353 (26)	49 (29)	30 (21)
**Parity**						
0 child	7372 (18)	1011 (17)	769 (17)	295 (22)	30 (18)	33 (24)
1 child	5763 (14)	850 (15)	702 (15)	209 (15)	29 (17)	21 (15)
2 children	14683 (37)	2130 (37)	1778 (38)	496 (37)	63 (38)	53 (38)
>=3 children	12307 (31)	1813 (31)	1400 (30)	353 (26)	45 (27)	33 (24)
**Age at first birth**						
nonparous or no term pregnancy	8147 (21)	1140 (20)	864 (19)	317 (24)	34 (20)	40 (29)
<25 y	16625 (42)	2758 (48)	2011 (44)	384 (29)	51 (31)	33 (24)
25-<30 y	8953 (23)	1182 (21)	1052 (23)	363 (27)	50 (30)	43 (31)
30-<35 y	4201 (11)	479 (8)	489 (11)	201 (15)	22 (13)	12 (9)
>=35 y	1706 (4)	176 (3)	186 (4)	81 (6)	9 (5)	12 (9)
**BMI at age 30**						
<18.5% kg/m2	1018 (3)	157 (3)	145 (3)	48 (4)	2 (1)	3 (2)
18.5–24.9 kg/m2	30242 (76)	4294 (74)	3464 (75)	946 (70)	113 (68)	91 (65)
25.0–29.9 kg/m2	6186 (16)	896 (16)	711 (15)	240 (18)	33 (20)	37 (26)
>=30.0 kg/m2	2376 (6)	425 (7)	294 (6)	116 (9)	19 (11)	9 (6)
**Use of hormonal birth control**						
Nonuser	6190 (16)	760 (13)	505 (11)	137 (10)	20 (12)	7 (5)
<10 y	22892 (57)	3538 (61)	2893 (62)	731 (54)	92 (55)	80 (57)
>=10 y	10841 (27)	1485 (26)	1234 (27)	482 (36)	56 (33)	53 (38)
**Use of hormonal replacement therapy**						
Never	23444 (58)	2866 (50)	2778 (60)	1231 (91)	134 (80)	127 (92)
Estrogen only	7579 (19)	1596 (28)	856 (18)	78 (6)	27 (16)	7 (5)
Combined estrogen and progestin	9109 (23)	1326 (23)	1017 (22)	44 (3)	7 (4)	4 (3)
**Migraine medication**						
No	N/A	1289 (22)	555 (12)	N/A	48 (29)	13 (9)
Yes	N/A	4506 (78)	4090 (88)	N/A	120 (71)	127 (91)
**Menopause**						
Premenopausal	12239 (30)	1354 (23)	1706 (37)	1163 (86)	110 (65)	116 (83)
Postmenopausal	26612 (66)	4132 (71)	2733 (59)	93 (7)	35 (21)	10 (7)
Premenopausal hysterectomy, with retained ovarian tissue	1303 (3)	321 (6)	212 (5)	95 (7)	23 (14)	14 (10)

^*^All age-dependent variables, *e*.*g*., menopausal status, were values at enrollment for Sister Study and values at the sistership-defined index age for Two Sister Study. The numbers for each variable do not add up to the total due to missingness.

**Table 2:** Multivariable adjusted hazard ratios for breast cancer.

Cancer Type	Study	Case Number (N)			Hazard Ratio (95% CI)		
		No migraine	Nonmenstrual migraine	Menstrually-related migraine	Any Migraine*	Nonmenstrual migraine*	Menstrually-related migraine*
Any	Sister	1701	246	169	0.98 (0.88,1.09)	1.03 (0.90,1.18)	0.91 (0.77,1.07)
	Two Sister	1157	139	122	0.97 (0.79,1.19)	0.97 (0.75,1.26)	0.97 (0.74,1.27)
	Combined	2858	382	291	0.98 (0.89,1.07)	1.02 (0.91,1.15)	0.92 (0.80,1.06)
Invasive	Sister	1220	189	124	1.03 (0.90,1.16)	1.10 (0.94,1.28)	0.94 (0.78,1.13)
	Two Sister	976	121	103	0.97 (0.78,1.21)	1.00 (0.75,1.32)	0.94 (0.70,1.27)
	Combined	2196	310	237	1.01 (0.90,1.13)	1.07 (0.93,1.22)	0.94 (0.80,1.10)
DCIS	Sister	412	41	34	0.73 (0.57,0.94)	0.73 (0.53,1.01)	0.73 (0.51,1.05)
	Two Sister	167	18	18	1.04 (0.61,1.79)	1.07 (0.51,2.24)	1.02 (0.49,2.11)
	Combined	479	59	52	0.77 (0.62,0.96)	0.75 (0.56,1.01)	0.79 (0.58,1.08)
ER+/PR+ invasive	Sister	730	110	80	1.05 (0.89,1.23)	1.08 (0.88,1.32)	1.01 (0.80,1.28)
	Two Sister	671	78	74	1.05 (0.79,1.37)	0.96 (0.68,1.36)	1.16 (0.79,1.69)
	Combined	1401	188	154	1.04 (0.9,1.19)	1.03 (0.86,1.23)	1.04 (0.86,1.27)
ER−/PR− invasive	Sister	163	30	13	1.01 (0.72,1.42)	1.29 (0.87,1.91)	0.68 (0.39,1.20)^#a^
	Two Sister	196	31	19	0.83 (0.52,1.33)	1.34 (0.7,2.57)	0.54 (0.28,1.02)^#b^
	Combined	359	61	32	0.96 (0.73,1.26)	1.30 (0.93,1.81)	0.63 (0.42,0.96)^#c^
ER−/PR−/HER2− invasive	Sister	111	20	9	1.05 (0.7,1.58)	1.34 (0.84,2.14)	0.70 (0.35,1.39)
	Two Sister	130	18	11	0.79 (0.43,1.45)	1.37 (0.58,3.24)	0.50 (0.22,1.12)
	Combined	209	38	18	0.98 (0.70,1.37)	1.33 (0.89,1.99)	0.64 (0.38,1.07)^#d^

^#a–#^d We tested for heterogeneity between menstrual and nonmenstrual migraine and observed *P *≤ 0.05 for a–d: ^#a^*P *= 0.05; ^#b^*P *= 0.04; ^#c^*P *= 0.005; ^#d^*P *= 0.02.

^*^Hazard ratios for breast cancer with nonmigraine as the reference group from Cox regression adjusting for race, age at menarche, BMI at age 30, age at first birth and menopause status. Study was adjusted for in the combined analysis through stratification.

**Table 3:** Case only analysis for heterogeneity in risk for different breast cancer subcategories.

Case only comparison	Study&	Cancer subcategory	Case Number (N)			Odds Ratio (95% CI)	
			No Migraine	Nonmenstrual migraine	Menstrual migraine	Nonmenstrual migraine*	Menstrual migraine*
Invasive ER+ versus ER−	Sister	ER+	934	131	97	1	1
		ER−	181	31	16	1.14 (1.75,0.74)	0.77 (1.35,0.44)
	Two Sister	ER+	754	86	83	1	2
		ER−	214	34	19	1.33 (2.08,0.86)	0.84 (1.43,0.49)
	Combined	ER+	1688	217	180	1	3
		ER−	395	65	35	1.23 (1.67,0.9)	0.79 (1.16,0.54)
Invasive** ER/PR status	Sister	ER+/PR+	730	110	80	1	1
		ER+/PR−	157	20	13	0.94 (0.63,1.41)	0.91 (0.59,1.42)
		ER−/PR−	163	31	13	1.27 (0.90,1.80)	0.71 (0.46,1.09)
	Two Sister	ER+/PR+	671	77	75	1	1
		ER+/PR−	72	7	7	0.90 (0.50,1.62)	1.02 (0.56,1.84)
		ER−/PR−	196	31	19	1.26 (0.90,1.76)	0.85 (0.59,1.24)
	Combined#	ER+/PR+	1401	187	155	1	1
		ER+/PR−	229	27	20	0.92 (0.66,1.27)	0.94 (0.67,1.33)
		ER−/PR−	359	62	32	1.26 (0.99,1.60)	0.78 (0.59,1.03)
DCIS versus Invasive	Sister	DCIS	1220	189	124	1	1
		Invasive	445	51	44	1.33 (0.96,1.86)	1.08 (0.75,1.57)
	Two Sister	DCIS	976	121	103	1	1
		Invasive	170	18	18	1.19 (0.70,2.02)	1.06 (0.62,1.80)
	Combined	DCIS	2196	310	227	1	1
		Invasive	615	69	62	1.28 (0.96,1.69)	1.05 (0.78,1.42)

^*^Odds ratios from logistic regression adjusting for race, age at menarche, age at first birth, menopause status, BMI at age 30 and age at breast cancer diagnosis.

^**^There were few in the ER−/PR+ category and those events were treated as censoring times in the Sister Study and affected families were eliminated in the Two Sister Study analysis.

^&^In the merged data analysis a dummy variable for study was also adjusted in the model.

^#^We tested for heterogeneity between menstrual and nonmenstrual migraine and observed *P* = 0.044.
