# Corrigendum: Migraine and possible etiologic heterogeneity for hormone-receptor-negative breast cancer

**DOI:** 10.1038/srep17238

**Published:** 2016-01-07

**Authors:** Min Shi, Lisa A. DeRoo, Dale P. Sandler, Clarice R. Weinberg

Scientific Reports 5: Article number: 1494310.1038/srep14943; published online: 10122015; updated: 01072016.

In this Article, Dale P. Sandler is incorrectly affiliated with ‘Department of Global Public Health and Primary Care, University of Bergen, Bergen, Norway’. The correct affiliation is listed below:

Epidemiology Branch, NIEHS, NIH, DHHS, Research Triangle Park.

